# Comprehensive spatial profiling reveals transitional microbiome dynamics and microbial heterogeneity in pediatric adenoid hypertrophy

**DOI:** 10.1002/imo2.70101

**Published:** 2026-05-10

**Authors:** Kaining Chen, Xin Wan, Wenli Guo, Hongyan Yu, Yuantao Tong, Huiwen Ke, Junjie Fang, Xilian Luo, Di Che, Chao Hou, Xu Mei, Bingxue Hu, Yuehong Liu, Xiaoxiao Yu, Daoman Xiang, Wensi Chen, Yingjia Zeng, Yufen Xu, Jun Shen, Qiuxia Chen, Lei Pi, Lanyan Fu, Huazhong Zhou, Bingshao Liang, Meng Zhang, Yifan Dong, Yanqiu Chen, Wenhao Zhou, Xiaoqiong Gu

**Affiliations:** ^1^ Guangzhou Women and Children's Medical Center Guangzhou Medical University Guangzhou China; ^2^ Department of Laboratory Medicine the Second Clinical College of Guangzhou University of Chinese Medicine Guangzhou China; ^3^ Center for Infectious Diseases Vision Medicals Co., Ltd Guangzhou China; ^4^ Department of Emergency Medicine The Fifth Affiliated Hospital of Guangzhou Medical University Guangzhou China

## Abstract

This study characterizes the upper respiratory microbiome in 276 children (101 Adenoid hypertrophy (AH) 119 Adenotonsillar hypertrophy (ATH), 11 Tonsil hypertrophy (TH), and 45 healthy controls by analyzing 1149 samples across five distinct niches: nasopharyngeal swabs (NS), adenoid swab (AS), and tonsil swabs (TS), plus adenoid tissues (AT) and tonsil tissues (TT). Utilizing a workflow of 16S rRNA sequencing, qPCR, and metagenomics, we identified significant spatial heterogeneity and niche‐specific dominance, such as *Staphylococcus* in the nasopharynx and *Streptococcus* on mucosal surfaces. AT exhibited a transitional microbiome with intermediate composition and unique *Ralstonia* enrichment. Machine learning models demonstrate that AT microbial profiles (area under curve, AUC = 0.752) offer superior predictive power for AH severity compared to surface swabs. SHAP analysis identified key contributing genera, such as *Roseburia* and *Flavobacterium*, as primary drivers of these predictive outcomes. These findings highlight how tissue‐infiltrating microbial dynamics drive chronic inflammation and pediatric hypertrophy.

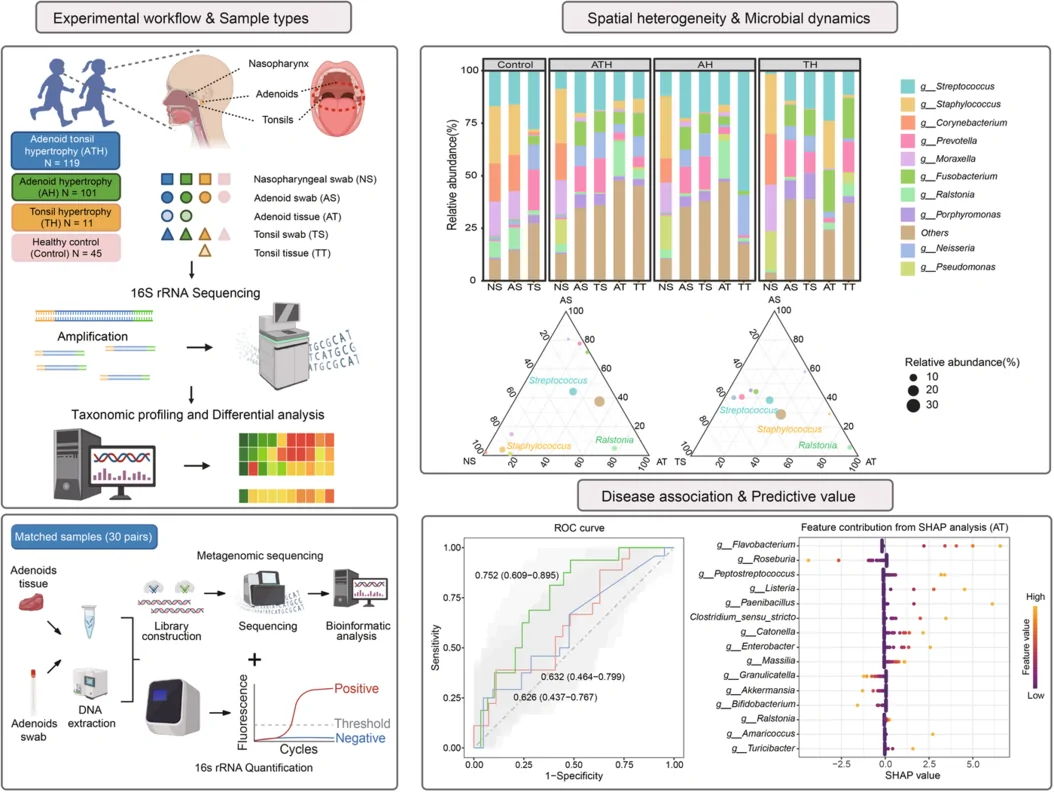

To the Editor,

Adenoid hypertrophy (AH) and adenotonsillar hypertrophy (ATH) are leading causes of pediatric upper airway obstruction, accompanied by comorbidities such as obstructive sleep apnea, chronic rhinosinusitis, and otitis media, which affect the health of approximately 34–46% of children worldwide [1, 2, 3]. The pathogenesis of AH and ATH involves a complex interplay of immunological dysregulation, genetic predispositions, and environmental factors such as recurrent infections, gastroesophageal reflux, and passive smoking, which exacerbate microbial‐immune imbalances and promote hypertrophic changes [2, 3, 4]. Dysbiosis of the upper respiratory tract microbiota plays an important role in the development of AH/ATH [5, 6, 7]. Previous research highlights microbial signatures such as elevated alpha diversity in tonsillar hypertrophy (TH) and pathogen enrichment (e.g., *Haemophilus*, *Streptococcus*) in AH, but these studies have some limitations, including fragmented sampling, small cohorts, and inadequate spatial profiling across niches such as the nasopharynx, surfaces, and tissues [8, 9, 10]. This hinders understanding of how microbiota dynamics drive chronic inflammation and tissue invasion. Thus, it is important to comprehensively investigate the respiratory microbial community structures in children with AH and ATH using larger cohorts and more diverse sample types.

To bridge these gaps, we profiled 1149 samples (NS: nasopharyngeal swabs, AS: adenoid swabs, TS: tonsil swabs, AT: adenoid tissues, TT: tonsil tissues) from 276 children (101 AH, 119 ATH, 11 TH, 45 controls). 16S rRNA and metagenomic sequencing revealed the overall compositional profiles of respiratory microbiota in children with AH and highlighted distinct microbial differences across sample type subgroups. Functional and network analyses further elucidated potential mechanisms underlying pathogen‐host interactions.

## 1 MICROBIAL PROFILING OF PEDIATRIC RESPIRATORY AND ADENOID TISSUES UNVEILS SPATIAL HETEROGENEITY IN MICROBIAL DIVERSITY AND COMPOSITION

Our study cohort comprised 276 children, including 134 males (58.0%) in the AH, TH and ATH group (mean age: 76.9 ± 3.6 months) and 28 males (62.2%) in the healthy control group (mean age: 64.7 ± 10.8 months) (Figure 1A and Table S1). By performing 16S rRNA sequencing on 1149 clinical specimens, including NS, AS, AT, TS, and TT, we obtained 89,624,391 high‐quality sequences. The sequencing depth averaged 78,002 reads per sample with 99.32% sample coverage (Table S2). Rarefaction curve analysis confirmed sufficient sequencing depth for characterizing microbial communities in both respiratory secretions and tissue specimens (Figure S1). Taxonomic classification revealed 17 phyla, 26 classes, 71 orders, 122 families, and 230 genera. After removing contaminants and retaining taxa with ≥1% abundance in at least 10% of samples, 25 genera were retained for subsequent analyses (Table S3).

**Figure 1:**
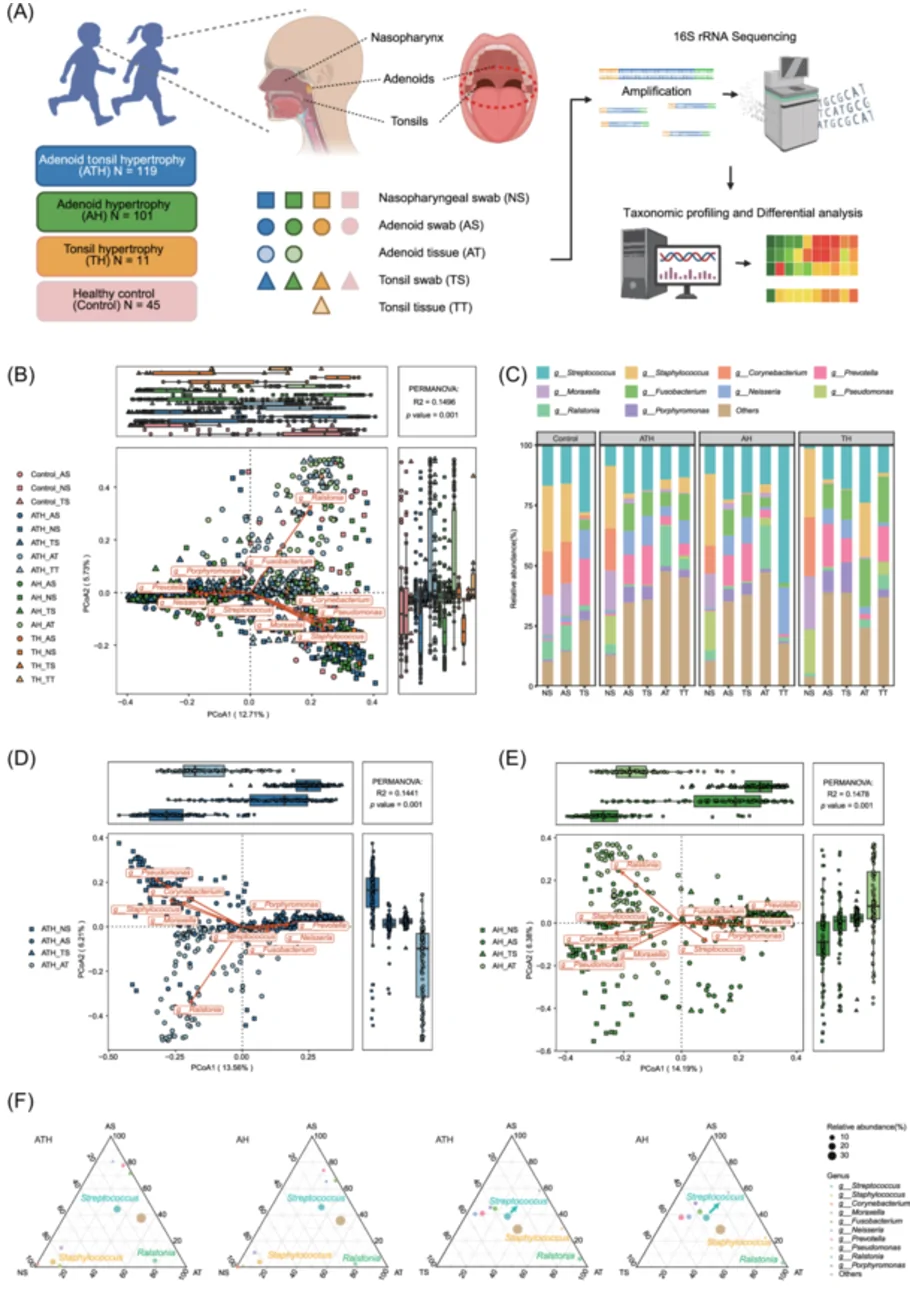
Taxonomic profiling and differential analysis of nasopharyngeal, adenoid, and tonsil microbiota. (A) Study design: adenotonsillar hypertrophy (ATH, *N* = 119), adenoid hypertrophy (AH, *N* = 101), tonsil hypertrophy (TH, *N* = 11), and healthy controls (Control, *N* = 45). Sample types include nasopharyngeal swab (NS), adenoid swab (AS), adenoid tissue (AT), tonsil swab (TS), and tonsil tissue (TT). Experimental workflow outlines 16S rRNA sequencing, amplification, taxonomic profiling, and differential analysis. (B) Principal coordinate analysis (PCoA) plots comparing microbial communities across groups and sample types. Group symbols: ATH (circles), AH (squares), TH (triangles), Control (diamonds). Sample type colors: NS (blue), AS (orange), AT (green), TS (purple), TT (red). PERMANOVA analysis reveals significant differences between groups. (C) Taxonomic composition bar plots showing relative abundances of bacterial genera (color‐coded legend) within each clinical group. (D, E) PCoA of microbiota across ATH and AH subgroups (NS, AS, TS, AT), illustrating intergroup separation (PERMANOVA: *R*² = 0.144, *p*‐value = 0.001; *R*² = 0.1478, *p*‐value = 0.001). Dominant genera (e.g., *Staphylococcus*, *Streptococcus*, *Corynebacterium*, *Moraxella*) are annotated with orange arrows. (F) Ternary plots illustrating the relative abundance of *Streptococcus*, *Staphylococcus*, and *Ralstonia* in various groups.

Principal coordinate analysis (PcoA) demonstrated significant beta‐diversity between ATH/AH/TH patients and controls (PERMANOVA: *R*² = 0.1496, *p‐*value = 0.001), with distinct microbial clustering patterns separating respiratory tract communities from AT microbiota (Figure 1B). Beta‐diversity showed that concurrent ATH did not significantly alter microbial community structures in respiratory secretions or AT (Figure S2; *p*‐value > 0.05). Comparative taxonomic analysis revealed niche‐specific dominance patterns: *Staphylococcus* in NS (ATH: 5.62% [0.61–47.89], AH: 7.24% [0.83–60.38], TH: 13.57% [6.49–35.09], Control: 8.08% [1.00–45.49]), *Streptococcus* in AS (ATH: 17.17% [6.93–29.68], AH: 17.80% [7.21–32.34], TH: 12.03% [6.98–20.00], Control: 3.72% [0.65–24.41]), and *Ralstonia* in AT (ATH: 6.36% [1.00–26.08], AH: 7.36% [1.31–28.76]) (Figure 1C). Relative abundance of the top 10 genera also revealed that AT exhibited combined NS/AS signatures with unique *Ralstonia* predominance (Figure 1C, Figure S3A), suggesting potential bidirectional microbial influences from these sampling sites. Beta‐diversity of AT displayed intermediate clustering between NS and AS (Figure 1D,E). These observations highlight significant spatial variations in microbial diversity and composition, with distinct genus dominances across niches that differentiate disease groups from controls.

## 2 TRANSITIONAL MICROBIOME FEATURES AND TAXONOMIC ENRICHMENTS IN ADENOID TISSUES

To investigate the relationship between the internal microbiome of AT and respiratory tract surface microbiota in children with ATH and AH, we compared the dominant genera *Streptococcus* and *Staphylococcus* across different sample types (Figure S3B–G). Notably, the relative abundance of *Streptococcus* was significantly elevated in AS and TS samples from both ATH and AH groups compared to NS and AT samples (Figure S3B,C; Wilcoxon rank‐sum test, *p*‐value < 0.01). No significant intergroup differences were observed in AT samples between ATH and AH cohorts (Figure S3D; *p*‐value > 0.05). Conversely, *Staphylococcus* demonstrated significantly higher relative abundance in NS and AT samples compared to AS/TS specimens (Figure S3E,F; *p*‐value < 0.01), with NS samples showing the highest levels of *Staphylococcus* in both ATH and AH patients. Again, no significant differences emerged between ATH and AH groups in AT samples (Figure S3G; *p*‐value > 0.05).

LefSe analysis confirmed the niche‐specific microbial signatures, suggesting that the combined signatures of swabs constituted AT communities (LDA ≥ 4, Figure S3H,I). Ternary plot analysis further revealed that *Staphylococcus* primarily originated from NS, *Streptococcus* was enriched in AS/TS samples, and *Ralstonia* was significantly enriched in AT (Figure 1F). Furthermore, we compared the relative abundances of key microorganisms in NS samples among disease groups. No significant difference was observed in dominant genera (*Staphylococcus and Streptococcus*), but subdominant genera (*Haemophilus, Pseudomonas*, and *Ralstonia*) demonstrated statistically elevated abundances in AH/ATH groups compared to Controls (Figure S4; Wilcoxon rank‐sum test, *p*‐value < 0.05). The results demonstrate transitional microbiome patterns in AT, with intermediate compositions and specific taxonomic enrichments that distinguish disease states from healthy controls.

## 3 DIVERGENT MICROBIAL NETWORK TOPOLOGIES AND FUNCTIONAL PATHWAYS IN ADENOID TISSUES

Further microbial interactions through the network exhibited pronounced reorganization under ATH and AH, marked by divergent network architectures and niche‐specific dominance of genus. AS networks exhibited denser clustering with a prominent large module (M1, *Staphylococcus*), while AT networks were relatively sparse and fragmented, consisting of small and loosely connected modules (Figure S5A). Structural divergence was quantified using the dissimilarity index *D*, referenced against AT networks (Figure S5B). AS exhibited the highest divergence from AT (*D* ≈ 0.4 for AH, *D* ≈ 0.3 for ATH), with NS and TS demonstrating intermediate (*D* ≈ 0.2) and minimal (*D* ≈ 0.1) divergence, respectively. Module composition further underscored these differences (Figure S5C). Across groups, M1 (*Staphylococcus*) was consistently the largest module, particularly in AH_AS and AH_AT, while M2 (*Streptococcus*) dominated in NS and TS. Network density metrics reinforced distinct microbial interaction patterns shaped by sample type and disease phenotype, with AH networks exhibiting greater connectivity than ATH, and AS/AT displaying more extensive microbial crosstalk than NS/TS samples (Figure S5D,E). NetMoss analysis identified key genera driving these interaction differences (Figure S5F,G).

The predicted functional profiling of microbial communities revealed distinct correlations between KEGG pathways and microbial abundances across anatomical sampling sites. We found that pathways associated with purine, amino acid, and fatty acid metabolism, such as ko00230, ko00071, ko00280, and ko00380, demonstrated significant correlations with the relative abundance of three dominant genera (*Staphylococcus, Haemophilus, and Streptococcus*) (Spearman |*R* | > 0.3, adjusted *p‐*value < 0.05; Figure S6A). In addition, tissue samples exerted obviously divergent enriched abundance compared to the swab samples, and nasopharyngeal samples differed from adenoid samples (Figure S6A). Further comparative analysis across sample types revealed consistent spatial stratification for illustrated pathways (Figure S6B–F). AT and NS exhibited higher median relative abundances for four pathways in AH and ATH, while surface swabs (AS/TS) showed substantially lower values (*p*‐value < 0.0001). Despite quantitative variations, all pathways followed identical spatial hierarchies (NS ≈ AT > AS ≈ TS).

## 4 EXPLORING THE PREDICTIVE VALUE OF MICROBIOME COMPOSITION IN ADENOID HYPERTROPHY SEVERITY

To further validate the microbial composition at the species level, we performed metagenomic sequencing and 16S rRNA quantitative PCR (qPCR) on matched AS and AT samples from 30 patients (Figure 2A). *Staphylococcus aureus* (*S. aureus*), *Haemophilus influenzae*, *Streptococcus pneumoniae* (*S. pneumoniae*), and *Moraxella catarrhalis* were identified as the four most abundant microbial species across AS and AT samples (Figure 2B). Hematoxylin and eosin histological staining combined with FISH experiments, and bacterial cultures further confirmed the presence of viable *S. aureus* in both the superficial and deep tissue compartments (Figure S7). Phylogenetic clustering revealed distinct strain distribution patterns: *S. aureus* isolates exhibited high diversity without dominant lineages, with GCF_018987365 showing the highest prevalence (3/31, 9.7%; Figure S8). Conversely, *S. pneumoniae* demonstrated significant clonal predominance, with GCF_900475305 accounting for 48.6% of isolates (17/35; Figure S8). No distributional differences of AH grades among strains were observed. Intriguingly, both *S. aureus* and *S. pneumoniae* strains from AS samples showed closer phylogenetic relationships to their AT counterparts within the same patients compared to inter‐individual comparisons (Figure S8).

**Figure 2:**
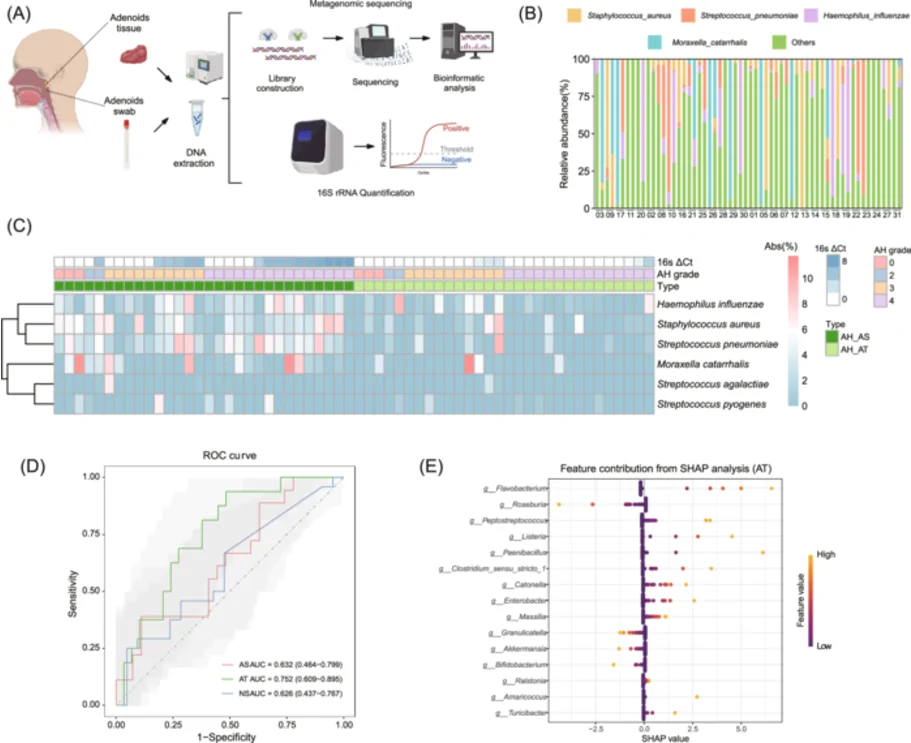
Microbial profiling and association with adenoid hypertrophy grade. (A) Schematic workflow of DNA extraction from adenoid tissue and swabs, 16S rRNA quantification, metagenomic sequencing, and bioinformatic analysis. (B) Species relative abundance bar plot based on metagenomic sequencing across samples based on metagenomic sequencing. Paired AS (left bar) and AT (right bar) values for each of the 30 subjects. Each subject is represented by two adjacent bars; the 60 bars collectively show the comparison between the two sample types across all paired samples. (C) Heatmap displaying relative abundance of bacterial species across sample types and disease grades. (D) Area under the receiver operating characteristic curve (AUC) values for the predictive performance of the three models in classifying AH severity, along with their 95% confidence intervals (gray). (E) Feature contribution distribution in the AT (SHAP values), illustrating the contributions of different microbial genera to the overall predictive capability. Negative SHAP values indicate contributions toward classifying samples as mild (grade I–III) symptoms, while positive values indicate contributions toward classifying samples as severe (grade IV) symptoms. AH, adenoid hypertrophy; AS, adenoid swab; AT, adenoid tissue.

To explore potential associations between microbial species and disease severity, we assessed the frequency of microbial detection at different AH grades. No significant differences in species occurrence frequencies were observed among different AH severity groups for both AS and AT samples (*p*‐value > 0.05; Figure 2C and Table S4). We next identified differential taxa that might hold predictive value for distinguishing mild (grade I–III) from severe (grade IV) AH (Figure 2D). Notably, the AT sample model achieved an AUC of 0.752, while the microbial communities in AS and NS samples exhibited limited discriminatory power for AH (AUC = 0.632 and 0.626, respectively). These findings suggested that the microbial composition within AT exhibited discernible differences across AH grades, whereas the microbial profiles on oral surfaces showed minimal variation between severity degrees of AH. SHapley Additive exPlanations (SHAP) analysis of AT samples indicated that a high abundance of the genus *Roseburia* was strongly associated with mild symptoms (Figure 2E). Conversely, elevated loads of *Flavobacterium*, *Massilia*, *Catonella*, *Listeria*, and *Enterobacter* were more prevalent in samples from severe AH cases. Our study provided a comprehensive spatial profiling of the upper airway microbiome in pediatric AH and ATH, revealing transitional microbiome dynamics, spatial heterogeneity, and microbial signatures that distinguish diseased states from healthy controls. Core communities in AT and mucosal surfaces were dominated by *Firmicutes*, *Proteobacteria*, and *Bacteroidetes*, consistent with prior reports [8, 9, 11, 12, 13]. Notably, we observed niche‐specific dominance patterns, such as *Streptococcus* enrichment in AS and TS, *Staphylococcus* in NS, and intermediate communities in AT, suggesting a transitional microbiome, where surface microbes may infiltrate deeper tissues, fostering chronic inflammation and hypertrophy. Discrepancies between our results and previous studies highlight methodological, regional, and cohort‐related variations. Traditional culture‐based approaches often prioritize aerobic pathogens but overlook anaerobes or intracellular bacteria due to cultivation biases [14]. In contrast, 16S rRNA sequencing uncovers anaerobic biofilm communities in hypoxic adenoid crypts, implying metabolic cross‐feeding that sustains dysbiosis [10]. Regional differences further contribute to heterogeneity: European cohorts show *Veillonella* dominance [15], while Asian populations exhibit *Haemophilus* enrichment [16], potentially influenced by environmental exposures such as pollution or diet. Our findings extend prior research, which often relied on limited sampling [17, 18], by highlighting AT as a transitional niche exhibiting intermediate compositions influenced by both NS and AS microbiotas. Strain‐resolved metagenomics confirmed intra‐patient migration of *S. aureus* and *S. pneumoniae* from nasal reservoirs to adenoid tissues, challenging the unidirectional “pathogen reservoir” model [2]. This spatial heterogeneity underscores the role of microbial migration and adaptation in driving chronic inflammation and hypertrophic changes.

Network and functional analyses further deepen our understanding of microbial interactions in AH/ATH pathogenesis. Divergent network topologies showed denser connectivity in AT compared to NS/TS, with AS exhibiting the highest divergence from AT, indicating niche‐specific reorganization. Functional profiling links dominant genera to pathways enriched in NS and AT compared to surface swabs. These patterns imply that dense interactions enhance metabolic activities contributing to immune dysregulation and inflammation [19]. Spatial stratification of network and functional pathways in this study also underscores progressive dysbiosis gradients, where tissue microbiomes mirror nasopharyngeal influences but amplify pathogenic functions. Microbial interactions determined chronic airway disease exacerbation risk, which cannot be appreciated by a single microbial group [20]. The niche‐specific discrepancy may stem from the microbe's interactome, rather than a single microbial group.

To our knowledge, this is the largest pediatric AH/ATH cohort with paired respiratory‐tissue samples and the first comprehensive microbiome atlas for East Asian children, addressing gaps in spatial profiling. Our spatial profiling reveals transitional communities of tissue samples with intermediate composition, dense microbial network, and metabolism‐associated pathway shifts that sustain pediatric AH/ATH. Shared dysbiosis patterns across AH/ATH emphasize the microbiota's pivotal role beyond single genus. Future studies should incorporate longitudinal tracking, multi‐omics integration, and interventions targeting key microbial networks to develop microbiome‐based therapies for AH/ATH, ultimately improving pediatric respiratory health outcomes.

## AUTHOR CONTRIBUTIONS

**Kaining Chen, Xin Wan**, and **Wenli Guo**: Methodology; investigation; formal analysis; writing—original draft. **Hongyan Yu** and **Yuantao Tong:** Data curation; validation. **Huiwen Ke, Xilian Luo**, and **Junjie Fang**: Software; visualization. **Di Che, Chao Hou, Xu Mei, Bingxue Hu, Yuehong Liu, Xiaoxiao Yu, Daoman Xiang, Wensi Chen, Yingjia Zeng, Yufen Xu, Jun Shen, Qiuxia Chen, Lei Pi, Lanyan Fu, Huazhong Zhou, Bingshao Liang, Meng Zhang**, and **Yifan Dong**: Resources; investigation; writing—review and editing. **Yanqiu Chen, Wenhao Zhou**, and **Xiaoqiong Gu:** Conceptualization; supervision; project administration; funding acquisition; writing—review and editing. All authors have read the final manuscript andapproved it for publication.

## CONFLICT OF INTEREST STATEMENT

The authors declare no conflicts of interest.

## ETHICS STATEMENT

The ethics application (No. [2023] 177A2025041100) was approved by the Research Ethics Committee of the Guangzhou Women and Children's Medical Center, Guangzhou Medical University. The studies were conducted in accordance with the local legislation and institutional requirements. Written informed consent for participation in this study was provided by the participants' legal guardians/next of kin. Written informed consent was obtained from the individual(s), and minor(s)' legal guardian/next of kin, for the publication of any potentially identifiable images or data included in this article.

## Supporting information


**Figure S1.** Rarefaction analysis of microbial community richness across experimental groups.**Figure S2.** Structural Divergence of Microbial Communities Across subgroups and sample types.**Figure S3.** Microbial relative abundance analysis of different groups.**Figure S4.** Differential Distribution of Commensal and Pathogenic Bacterial Genera between healthy control and adenoid hypertrophy patients.**Figure S5.** Network analysis across different sample types of AH and ATH patients.**Figure S6.** Comparative analysis of gene functional profiles across sample types and disease states.**Figure S7.** Microscopic characterization and microbiological isolation of Staphylococcus aureus in infected adenoid tissues.**Figure S8.** Hierarchical clustering analysis of adenoid hypertrophy severity profiles across patient cohorts.

**Table S1.** Demographic and Clinical Characteristics of Study Cohort.**Table S2.** Read count and quality control metrics of the 16S rRNA sequencing across study groups. **Table S3.** Taxonomic classification of OTUs (Operational Taxonomic Unit) for all samples.**Table S4.** Comparative analysis of metagenomic detection rates for selected bacterial species between mild and severe AH patients.

## Data Availability

The data that support the findings of this study are available from the corresponding author upon reasonable request. All the sequencing data have been deposited in the National Genomics Data Center, China National Center for Bioinformation/Beijing Institute of Genomics, under BioProject accession number: https://ngdc.cncb.ac.cn/bioproject/browse/PRJCA041507. Scripts are deposited at https://github.com/kathyyChen/microbiome/. Supplementary materials (methods, figures, tables, graphical abstract, slides, videos, Chinese translated version, and updated materials) could be found in the online DOI or iMetaOmics http://www.imeta.science/imetaomics/.
