# Supplementary material for: Comprehensive spatial profiling reveals transitional microbiome dynamics and microbial heterogeneity in pediatric adenoid hypertrophy
Source: IMetaOmics. 2026 May 10;3(2):e70101. doi: 10.1002/imo2.70101 (PMC13674403; doi:10.1002/imo2.70101)
Supplement: Supplementary file 1 [file IMO2-3-e70101-s001.docx]

**Supporting information to**

**Comprehensive spatial profiling reveals transitional microbiome dynamics and microbial heterogeneity in pediatric adenoid hypertrophy**

**Running title**: Transitional microbiome in pediatric adenoid hypertrophy

Kaining Chen^1#^, Xin Wan^1#^, Wenli Guo^1#^, Hongyan Yu^1^, Yuantao Tong^1^, Huiwen Ke^2^, Junjie Fang^1^, Xilian Luo^1^, Di Che^1^, Chao Hou^1^, Xu Mei^3^, Bingxue Hu^3^, Yuehong Liu^1^, Xiaoxiao Yu^1^, Daoman Xiang^1^, Wensi Chen^1^, Yingjia Zeng^1^, Yufen Xu^1^, Jun Shen^1^, Qiuxia Chen^4^, Lei Pi^1^, Lanyan Fu^1^, Huazhong Zhou^1^, Bingshao Liang^1^, Meng Zhang^1^, Yifan Dong^1^, Yanqiu Chen^1*^, Wenhao Zhou^1*^, Xiaoqiong Gu^1*^

^1^Guangzhou Women and Children’s Medical Center, Guangzhou Medical University, Guangzhou 510623, China.

^2^Department of Laboratory Medicine, the Second Clinical College of Guangzhou University of Chinese Medicine, Guangzhou 510120, China.

^3^Center for Infectious Diseases, Vision Medicals Co., Ltd, Guangzhou 510700, China.

^4^Department of Emergency Medicine, The Fifth Hospital of Guangzhou Medicine University, Guangzhou 510040, China.

^#^These authors contributed equally: Kaining Chen, Xin Wan, Wenli Guo.

*Correspondence: [270003833@qq.com](mailto:270003833@qq.com) (Yanqiu Chen), [zhouwenhao@fudan.edu.cn](mailto:zhouwenhao@fudan.edu.cn) (Wenhao Zhou), [guxiaoqiong@gwcmc.org](mailto:guxiaoqiong@gwcmc.org) (Xiaoqiong Gu)

**MATERIALS AND METHODS**

**Patients, sample collection and clinical characteristics**

In total, our study cohort recruited 276 children, including 101 pediatric patients with AH, 119 with ATH, 11 with TH, and 45 healthy controls with matched gender and age. For AH/TH patients, nasopharyngeal swabs, adenoidal surface swabs, tonsillar surface swabs, and adenoid tissue/tonsillar tissue samples were collected. ATH patients were collected nasopharyngeal swabs, adenoidal surface swabs, tonsillar surface swabs, adenoid tissue, and tonsillar tissue. Healthy controls only provided nasopharyngeal swabs, adenoidal surface swabs, and tonsillar surface swabs (Figure 1A). Detailed medical information was collected from the patients, including age, height, body weight, and BMI. Children affected by grade II–IV AH with inflammation, in consultation for adenoidectomy and tonsillectomy, were enrolled at the Department of Otorhinolaryngology of Guangzhou Women and Children's Medical Center, Guangzhou, China, between July 2022 and September 2023.

The inclusion criteria were grade I–IV AH with inflammation with persisting symptoms for at least 1 year, ages 1-14 years, and enrolment in the surgical wait list for adenoidectomy and tonsillectomy for recurrent inflammation. Adenoid size was assessed by flexible fiberoptic nasopharyngoscopy and the classification system for adenoid hypertrophy was implemented as previously described [1]. Specially, the choanal opening was graded by dividing the vertical height (superior choanal border to nasal floor) into four equal parts and estimating the proportion occupied by adenoid tissue. We applied the Cassano endoscopic classification [2], as updated and reaffirmed in a large clinical series [3]: Grade I ( ≤ 25%), tissue confined to the upper quarter with the choana largely open; Grade II ( ≤ 50%), tissue in the upper half with the Eustachian tube orifice fully visible; Grade III ( ≈ 75%), substantial obstruction with partial involvement of the tube orifice and Grade IV ( > 75%), near-total obstruction with tissue reaching the inferior choanal border and preventing visualization of the tube orifice. Exclusion criteria comprised: (1) previous adenoidectomy/adenotonsillar surgery; (2) obstruction primarily explained by structural lesions (e.g., choanal atresia/diaphragm or marked septal deviation) or chronic rhinosinusitis with nasal polyps; (3) systemic conditions including rheumatological disorders, autoimmune diseases, craniofacial dysmorphism syndromes, or neuromuscular pathologies; (4) concurrent infectious processes (both suppurative and non-suppurative presentations); (5) severe asthma; (6) morbid obesity (BMI ≥ 40 kg/m²); (7) recent antimicrobial therapy within 15 days preceding enrollment; (8) corticosteroid administration within 30 days prior to study initiation; (9) inability to obtain informed consent. Patients meeting any exclusion parameter were excluded from study participation [3−6].

**Microbial genomic DNA extraction and 16S rRNA amplicon sequencing**

Genomic DNA was extracted from nasopharyngeal swabs, adenoidal surface swabs, tonsillar surface swabs, and adenoid tissue/tonsillar tissue of AH/TH/ATH patients and Healthy Control, and adenoid along with non-template negative controls to address potential background DNA contamination (n = 10). Total microbial genomic DNA extraction was performed using QIAamp DNA Microbiome Kit (Qiagen catalog no. 51704) following the manufacturer’s protocol ^[7]^. The V3–V4 hypervariable region of the bacterial 16S rRNA was amplified using universal primers 341F: CCTACGGGNGGCWGCAG; 805R: GACTACHVGGGTATCTAATCC, 30 cycles, 95°C denaturation, 55°C annealing, 72°C extension. The 16S ribosomal RNA genes were sequenced with the Illumina NovaSeq6000 platform to generate 250-bp pair-end reads [8,9].

**16S rRNA-seq data processing, denoise, and species annotation**

The raw data obtained by 16S rRNA sequencing were first performed quality control: PhiX sequences were removed, and paired-end reads with Q scores ≥ 20 were joined using QIIME2 software [10]. The DADA2 method was used for noise reduction, which generates amplicon sequence variants (ASVs) equivalent to 100% similarity operational taxonomic units (OTUs) [11]. By applying QIIME2's classify-sklearn algorithm, a pretrained Naive Bayes classifier is used for species annotation of each ASV [12]. Based on the ASVs annotations and the feature tables of each sample, taxa abundance tables were obtained at the levels of kingdom, phyla, class, order, family, genus, and species. Genera not colonizing humans or associated with kitome contaminants were removed from further analysis [13,14]. For adenoid and respiratory 16S rRNA profiles, we employed a simple statistical method to identify and remove potential contaminant sequences in marker-gene and metagenomics data. Subsequently, the abundance of each taxon was analyzed, leading to the removal of low-abundance species from further consideration. We retained taxa that exhibited an abundance of at least 1% in at least 15% of samples [14]. To obtain specific species, the assignments of the retained ASVs were searched based on BLASTn to annotate species [10].

**Alpha and beta diversity**

Alpha diversity indicates the number of species present in a typical area or ecosystem. Here, alpha diversity was used to analyze the complexity of species richness and diversity for each sample based on normalized ASV abundances through calculating the Shannon index using the QIIME2 software [10]. Principal coordinates analysis (PCoA) of relative abundance profiles for 1157 samples at the genus level was conducted. The dimension ordination provided position values along an ordination axis and distances from the axis for samples of communities [15].

**Co-occurrence analysis**

For the co-occurrence network analysis, the bacterial correlations within different groups were computed based on the relative abundance of each genus using SparCC with 100 bootstraps to estimate the *p* value. The correlation values with *p* value < 0.05 were retained [16,17]. The co-occurrence networks of the eight microbiota communities were visualized using Gephi software (https://gephi.org/). The closeness and eigenvector of the nodes were calculated to measure node centrality in each network [18].

**Between-group variation analysis**

High-dimensional biomarkers were discovered by LEfSe (Linear discriminant analysis Effect Size) [19] using the parameter “linear discriminant analysis (LDA) score > 4. LEfSe identifies different features (such as ASVs, genus, or genes) most likely to explain differences between classes by combining standard tests for statistical significance with additional tests encoding biological consistency and effect relevance [14].

**Construction of predictive models**

To investigate the relationship between different microbial colonization patterns and the severity of adenoidal hypertrophy (AH), we developed prediction models based on microbial abundance data from different sample types to classify AH severity. AH patients with grade I, II, and III were defined as “mild” symptoms, while grade IV was defined as “severe” symptoms. Binary prediction models were constructed using LASSO-regularized generalized linear models [20]. The models were built using genus-level relative abundance data derived from 16S rRNA sequencing encompassing three sample types: adenoid swab (AS), adenoid tissue (AT), and nasal swab (NS). Initially, LEfSe was applied to identify 116 genera with significant differential distribution across sample type groups (AS: 36, AT: 69, NS: 10; LDA score > 2, *p* value < 0.05). Subsequently, for each genus, we integrated its abundance data with participant sex and age to construct multifactorial generalized linear models. Microbial genera demonstrating discriminatory potential for AH severity were selected for final model construction (*p* value < 0.2), yielding 6 genera for AS, 21 for AT, and 2 for NS. Separate binary prediction models were built for AS, AT, and NS sample types. After comparing random forest and generalized linear models, the elastic-net regression was chosen for its combined L1 and L2 regularization advantages, which enhance stability and feature selection in high-dimensional data. Using relative abundance data from the 29 selected genera across 152 samples, the dataset was randomly split into 70% for training and 30% for testing. Binary classification models were built with the glmnet package using 10-fold cross-validation. The final model was determined by the lambda value minimizing cross-validation error. Model performance was evaluated using the area under the receiver operating characteristic curve (AUC) on the test set. The 95% confidence interval for AUC was estimated using 2000 bootstrap repetitions.

**16s rRNA quantification**

One hundred nanograms of DNA per reaction was analyzed by qPCR on an Applied Biosystems 7500 instrument for bacterial 16S rRNA genes using TaqMan Gene Expression Mix and previously published methods [21]. The primers and probe for 16S rRNA gene were as follows: forward primer 16S-F(5'-ACTCCTACGGGAGGCAGCAGT-3'), reverse primer 16S-R(5'-ATTACCGCGGCTGCTGGC-3'), and probe (5'-HEX-AGGGACTGGAAGGCTTTACTGCTTC-3'BHQ116). Cycling parameters were as follows: 50°C 20 min; 95°C 10 min; 40 cycles of 95°C 15 sec, 58°C 1 min.

**Metagenome sequencing**

Sixty paired-matched samples were collected for metagenomic sequencing. After conducting library quality control, the different libraries were pooled based on their effective concentration and the desired data amount. The 5′-end of each library was phosphorylated and cyclized. Subsequently, loop amplification was performed to generate DNA nanoballs. Finally, these DNA nanoballs were loaded into a flow cell with DNBSEQ-T7 for sequencing [22,23].

**Metagenomic data analysis**

The data quality control process involved discarding paired reads if (a) Either read contained adapter contamination, (b) More than 10% of bases were uncertain in either read, and (c) The proportion of low-quality bases (Phred quality < 5) exceeded 50% in either read. This process was carried out to obtain clean data using Fastp (version 0.23.1) [24]. To construct the microbial genome database, pathogens and their genomes or assemblies were selected following the Kraken2 criteria for selecting representative assemblies for microorganisms (bacteria, viruses, fungi, protozoa, and other multicellular eukaryotic pathogens) from the NCBI Assembly and Genome databases (<https://benlangmead.github.io/aws-indexes/k2>). Strain-level annotation of *Streptococcus pneumoniae* and *Staphylococcus aureus* was used StrainGE with default parameters [25]. Strain database is built using high-quality reference genomes (NCBI RefSeq complete assemblies of each species) for a target species or genus. K-mer profiles (k-mer size: k) are generated via sliding-window genome traversal. To optimize computational efficiency, a minhash algorithm [26] retains 1% of k-mers with minimal hash values. Then, we identify sample strains by intersecting sample-derived k-mers (K_sample) with the species-specific database, excluding non-target k-mers. An iterative scoring system evaluates reference genomes against K_sample, selecting top candidates exceeding a threshold (θ=0.02 by default). Matched k-mers are iteratively removed to enable secondary strain detection ( ≤ 5 iterations). The process terminates if no candidate surpasses θ, ensuring sensitivity while minimizing false positives. This approach facilitates precise strain resolution and multi-strain detection in complex samples [25,26].

**Fluorescence in situ hybridization (FISH) analysis of biofilms**

A tissue section from each patient was first stained using a Periodic Acid Schiff (PAS) [21] staining kit (Sigma-Aldrich, USA) to confirm the preservation of the mucus layer according to the manufacturer's directions. Successive sections were then stained with the universal bacterial probe EUB338 for the presence of the presence of *S. aureus* in paraffin-embedded adenoid tissue sections (5′-GCT GCC TCC CGT AGG AGT-3′) [27] or with the following specific bacterial probes (all from Sigma-Aldrich, USA) for determining the microbial composition of the biofilms: *Staphylococcus aureus* (5′-GAA GCA AGC TTC TCG TCC G-3′) [28]. Slides were first deparaffinized with 2 x 5 minute incubations in xylene, then hydrated with 2 x 5 min in 100% ethanol, 2 x 5 min in 95% ethanol, and 5 min in 70% ethanol. Slides were then rinsed in TRIS buffer. Probes were applied to slides at a concentration of 2 μM in a volume of approximately 150 μL per slide in prewarmed hybridization buffer (900 mM NaCl, 20 mM Tris pH 7.5, 0.01% SDS). Slides were incubated at 46°C in a humid chamber for 1.5 hr (universal probe) or 2 hr (multiple probes), then washed 3 x 5 min in wash buffer (215 mM NaCl, 20 mM Tris pH 7.5, 5 mM EDTA). Slides were then counterstained with DAPI (diluted 1:10,000 in PBS) for visualization of nuclei for approximately 5 min at room temperature, then washed 3 x 5 min again with wash buffer. Slide coverslips were mounted with ProLong Gold antifade reagent (Life Technologies). Slides stained with the universal probe were imaged using a Zeiss LSM 510 META laser scanning microscope with LSM Zen imaging software at 400X (including 10X magnification of lens). Slides with substantial autofluorescence in the mucus layer and slides stained with multiple probes were imaged on a Zeiss LSM 780 META laser scanning microscope at 400X with LSM Zen imaging software with linear unmixing in the Microscope Core Facility in order to differentiate either autofluorescence from 16S rRNA gene universal probe signal or for differentiation of signal from multiple probes using reference spectra from pure cultures of representative bacteria, respectively [29].

**REFERENCES**

1. Brodsky, Linda. 1989. “Modern assessment of tonsils and adenoids.” *Pediatric Clinics of North America* 36: 1551−1569. https://doi.org/10.1016/s0031-3955(16)36806-7

2. Cassano, Pasquale, Matteo Gelardi, Michele Cassano, Marialuisa Fiorella, R. Fiorella. 2003. “Adenoid tissue rhinopharyngeal obstruction grading based on fiberendoscopic findings: a novel approach to therapeutic management.” *International Journal of Pediatric Otorhinolaryngology* 67: 1303−1309. https://doi.org/10.1016/j.ijporl.2003.07.018

3. Cassano, Michele, Eugenio De Corso, Valeria Fiore, Rossana Giancaspro, Antonio Moffa, Manuele Casale, Eleonora Maria Consiglia Trecca, Dario Antonio Mele, Pasquale Cassano, Matteo Gelardi. 2022. “Update of endoscopic classification system of adenoid hypertrophy based on clinical experience on 7621 children.” *Acta Otorhinolaryngol Italica* 42: 257−264. https://doi.org/10.14639/0392-100x-n1832

4. Del Chierico, Federica, Antonia Piazzesi, Ersilia Vita Fiscarelli, Maria Vittoria Ristori, Ilaria Pirona, Alessandra Russo, Nicoletta Citerà, et al. 2024. “Changes in the pharyngeal and nasal microbiota in pediatric patients with adenotonsillar hypertrophy.” *Microbiol Spectrum* 12: e0072824. https://doi.org/10.1128/spectrum.00728-24

5. Jo, Jae-Won, Sung Ktun Kim, Jae Yong Byun, Seok Min Hong, Bong-Soo Kim. 2025. “The association between the adenoid microbiome and chronic otitis media with effusion in children differs according to age.” *Frontiers in Cellular and Infection Microbiology* 15: 1660939. https://doi.org/10.3389/fcimb.2025.1660939

6. Feres, Murilo F.N., Juliana S. Hermann, Ana C. Sallum, Shirley Shizue Nagata Pignatari. 2014. “Radiographic adenoid evaluation--suggestion of referral parameters.” *Jornal de Pediatria (Rio J)* 90: 279−285. https://doi.org/10.1016/j.jped.2013.09.004

7. Wu-Woods, Natalie J., Jacob T. Barlow, Florian Trigodet, Dustin G. Shaw, Anna E. Romano, Bana Jabri, A. Murat Eren, Rustem F. Ismagilov. 2023. “Microbial-enrichment method enables high-throughput metagenomic characterization from host-rich samples.” *Nature Methods* 20: 1672−1682. https://doi.org/10.1038/s41592-023-02025-4

8. Gao, Chuqi, Xuantao Li, Xiaole Zhao, Peiyue Yang, Xiao Wang, Xiaoli Chen, Ning Chen, Feng Chen. 2022. “Standardized studies of the oral microbiome: From technology-driven to hypothesis-driven.” *iMeta* 1: e19. https://doi.org/10.1002/imt2.19

9. Lu, Hongye, Peihui Zou, Yifei Zhang, Qian Zhang, Zhibin Chen, Feng Chen. 2022. “The sampling strategy of oral microbiome.” *iMeta* 1: e23. https://doi.org/10.1002/imt2.23

10. Bolyen, Evan, Jai Ram Rideout, Matthew R. Dillon, Nicholas A. Bokulich, Christian C. Abnet, Gabriel A. Al-Ghalith, Harriet Alexander, et al. 2019. “Reproducible, interactive, scalable and extensible microbiome data science using QIIME 2.” *Nature Biotechnology* 37: 852−857. https://doi.org/10.1038/s41587-019-0209-9

11. Callahan, Benjamin J., Paul J. McMurdie, Michael J. Rosen, Andrew W. Han, Amy Jo A. Johnson, Susan P. Holmes. 2016. “DADA2: High-resolution sample inference from Illumina amplicon data.” *Nature Methods* 13: 581−583. https://doi.org/10.1038/nmeth.3869

12. Wang, Qiong, George M. Garrity, James M. Tiedje, James R. Cole. 2007. “Naive Bayesian classifier for rapid assignment of rRNA sequences into the new bacterial taxonomy.” *Applied and Environmental Microbiology* 73: 5261−5267. https://doi.org/10.1128/aem.00062-07

13. Moreno, Inmaculada, Iolanda Garcia-Grau, David Perez-Villaroya, Marta Gonzalez-Monfort, Mustafa Bahçeci, Marcelo J. Barrionuevo, Sagiri Taguchi, et al. 2022. “Endometrial microbiota composition is associated with reproductive outcome in infertile patients.” *Microbiome* 10: 1. https://doi.org/10.1186/s40168-021-01184-w

14. Wang, Tao, Penghao Li, Xue Bai, Shilin Tian, Maosen Yang, Dong Leng, Hua Kui, et al. 2024. “Vaginal microbiota are associated with in vitro fertilization during female infertility.” *iMeta* 3: e185. https://doi.org/10.1002/imt2.185

15. Wen, Tao, Guoqing Niu, Tong Chen, Qirong Shen, Jun Yuan, Yong-Xin Liu. 2023. “The best practice for microbiome analysis using R.” *Protein & Cell* 14: 713−725. https://doi.org/10.1093/procel/pwad024

16. Friedman, Jonathan, Eric J. Alm. 2012. “Inferring correlation networks from genomic survey data.” *PLOS Computational Biology* 8: e1002687. https://doi.org/10.1371/journal.pcbi.1002687

17. Lin, Lifeng, Xinzhu Yi, Haiyue Liu, Ruilin Meng, Saiqiang Li, Xiaomin Liu, Junhao Yang, et al. 2023. “The airway microbiome mediates the interaction between environmental exposure and respiratory health in humans.” *Nature Medicine* 29: 1750−1759. https://doi.org/10.1038/s41591-023-02424-2

18. Shannon, Paul, Andrew Markiel, Owen Ozier, Nitin S. Baliga, Jonathan T. Wang, Daniel Ramage, Nada Amin, Benno Schwikowski, Trey Ideker. 2003. “Cytoscape: a software environment for integrated models of biomolecular interaction networks.” *Genome Research* 13: 2498−2504. https://doi.org/10.1101/gr.1239303

19. Segata, Nicola, Jacques Izard, Levi Waldron, Dirk Gevers, Larisa Miropolsky, Wendy S. Garrett, Curtis Huttenhower. 2011. “Metagenomic biomarker discovery and explanation.” *Genome Biology* 12: R60. https://doi.org/10.1186/gb-2011-12-6-r60

20. Tay, J. Kenneth, Balasubramanian Narasimhan, Trevor Hastie. 2023. “Elastic Net Regularization Paths for All Generalized Linear Models.” *Journal of Statistical Software* 106: https://doi.org/10.18637/jss.v106.i01

21. Tong, Jia, Chengxu Liu, Paula Summanen, Huaxi Xu, Sydney M. Finegold. 2011. “Application of quantitative real-time PCR for rapid identification of Bacteroides fragilis group and related organisms in human wound samples.” *Anaerobe* 17: 64−68. https://doi.org/10.1016/j.anaerobe.2011.03.004

22. You, Hailong, Bin Yang, Huifang Liu, Wencai Wu, Fei Yu, Nan Lin, WenJiao Yang , et al. 2025. “Unravelling distinct patterns of metagenomic surveillance and respiratory microbiota between two P1 genotypes of Mycoplasma pneumoniae.” *Emerg Microbes Infect* 14: 2449087. https://doi.org/10.1080/22221751.2024.2449087

23. Diao, Zhenli, Huiying Lai, Dongsheng Han, Bin Yang, Rui Zhang, Jinming Li. 2023. “Validation of a Metagenomic Next-Generation Sequencing Assay for Lower Respiratory Pathogen Detection.” *Microbiology Spectrum* 11: e0381222. https://doi.org/10.1128/spectrum.03812-22

24. Chen, Shifu, Yanqing Zhou, Yaru Chen, Jia Gu. 2018. “fastp: an ultra-fast all-in-one FASTQ preprocessor.” *Bioinformatics* 34: i884−i890. https://doi.org/10.1093/bioinformatics/bty560

25. van Dijk, Lucas R., Bruce J. Walker, Timothy J. Straub, Colin J. Worby, Alexandra Grote, Henry L. Schreiber IV, Christine Anyansi, et al. 2022. “StrainGE: a toolkit to track and characterize low-abundance strains in complex microbial communities.” *Genome Biology* 23: 74. https://doi.org/10.1186/s13059-022-02630-0

26. Ondov, Brian D., Todd J. Treangen, Páll Melsted, Adam B. Mallonee, Nicholas H. Bergman, Sergey Koren, Adam M. Phillippy. 2016. “Mash: fast genome and metagenome distance estimation using MinHash.” *Genome Biology* 17: 132. https://doi.org/10.1186/s13059-016-0997-x

27. Ide, Keigo, Yohei Nishikawa, Toru Maruyama, Yuko Tsukada, Masato Kogawa, Hiroki Takeda, Haruka Ito, et al. 2022. “Targeted single-cell genomics reveals novel host adaptation strategies of the symbiotic bacteria Endozoicomonas in Acropora tenuis coral.” *Microbiome* 10: 220. https://doi.org/10.1186/s40168-022-01395-9

28. Armbruster, Catherine R., Kelvin Li, Megan R. Kiedrowski, Anna C. Zemke, Jeffrey A. Melvin, Jennifer Moore, Sarah Atteih, et al. 2022. “Low Diversity and Instability of the Sinus Microbiota over Time in Adults with Cystic Fibrosis.” *Microbiology Spectrum* 10: e0125122. https://doi.org/10.1128/spectrum.01251-22

29. Drewes, Julia L., James R. White, Christine M. Dejea, Payam Fathi, Thevambiga Iyadorai, Jamuna Vadivelu, April C. Roslani, et al. 2017. “High-resolution bacterial 16S rRNA gene profile meta-analysis and biofilm status reveal common colorectal cancer consortia.” *NPJ Biofilms Microbiomes* 3: 34. https://doi.org/10.1038/s41522-017-0040-3

**Figure S1 Rarefaction analysis of microbial community richness across experimental groups.** The curve plots demonstrate the relationship between sequencing depth (x-axis: 0-90,000 sequences) and observed Operational Taxonomic Units (OTUs, y-axis: 0-100) for 16 experimental groups, including adenoid hypertrophy (AH), adenotonsillar hypertrophy (ATH), tonsillar hypertrophy (TH), and control cohorts: nasopharyngeal swabs (NS), adenoid swabs (AS), tonsil swabs (TS), adenoid tissues (AT), tonsil tissue (TT). Each colored line represents a unique sample group (e.g., AH_AS: adenoid swabs of AH patients).

**Figure S2 Structural Divergence of Microbial Communities Across subgroups and sample types.** (A-D) comparing microbiota across experimental groups, with PERMANOVA statistics indicating significant intergroup variation in panel A (R² = 0.0241, *p* value = 0.004) and non-significant trends in panels B-D (*p* value > 0.05). Scatterplots (above) show microbial community clustering by group, while right-side boxplots visualize principal coordinate distributions. Dominant genera (e.g., *Staphylococcus*, *Streptococcus*, *Corynebacterium*, *Moraxella*) are annotated with directional arrows. Adenoid hypertrophy (AH), adenotonsillar hypertrophy (ATH), nasopharyngeal swabs (NS), adenoid swabs (AS), tonsil swabs (TS), adenoid tissues (AT), tonsil tissue (TT).

**Figure S3** **Microbial relative abundance analysis of different groups.** (A) The relative abundance of bacterial genera across ATH and AH groups is shown by bar plots. Each bar represents a sample, and colors indicate different genera. (B-G) Violin plots showing the relative abundance of *Streptococcus* and *Staphylococcus* across groups. Statistical significance is indicated by asterisks (ns, not significant; *: *p* value < 0.05; **: *p* value < 0.01; ***: *p* value < 0.001; ****: *p* value < 0.0001). (H and I) LDA (Linear Discriminant Analysis) effect size plots, where each dot represents a bacterial genus, and the size of the circle correlates with the relative abundance. Colors represent different subgroups, including ATH_NS, ATH_AT, ATH_TS, AH_NS, AH-TS, and AH_AT. Adenoid hypertrophy (AH), adenotonsillar hypertrophy (ATH), nasopharyngeal swabs (NS), adenoid swabs (AS), tonsil swabs (TS), adenoid tissues (AT), tonsil tissue (TT).

**Figure S4 Differential Distribution of Commensal and Pathogenic Bacterial Genera between healthy control and adenoid hypertrophy patients.** (A-F) It represents taxonomic profiles for *Staphylococcus* (A), *Haemophilus* (B), *Moraxella* (C), *Streptococcus* (D), *Pseudomonas* (E), and *Ralstonia* (F). Violin plots with overlaid data points show distribution density (kernel probability estimation) and raw measurements. Central horizontal lines denote median values. Statistical comparisons were performed using pairwise Wilcoxon rank sum tests. Significance levels: ns = not significant (*p* value > 0.05), *: *p* value < 0.05; **: *p* value < 0.01; ***: *p* value < 0.001; ****: *p* value < 0.0001. Adenoid hypertrophy (AH), adenotonsillar hypertrophy (ATH), nasopharyngeal swabs (NS), adenoid swabs (AS), tonsil swabs (TS), adenoid tissues (AT).

**Figure S5 Network analysis across different sample types of AH and ATH patients.** (A) Representative microbial interaction networks: Microbial structures are color- and shape-coded: M1 (green circles), M2 (orange triangles), M3 (purple squares), M4 (red diamonds), M5 (pink hexagons), M6 (brown stars). Gray lattice backgrounds represent substrate surfaces. (B) Bar charts comparing the dissimilarity index D (derived from AH_AT) for groups AH_NS, AH_TS, AH_AS, ATH_NS, ATH_TS, and ATH_AS. Higher D values indicate greater network structural divergence. (C) Bubble plot of module sizes (M1–M6), color-coded by module identity. (D) Bar chart displaying the number of edges (connections) in networks across groups AH_NS, AH_TS, AH_AS, AH_AT, ATH_NS, ATH_TS, ATH_AS, and ATH_AT. (E) Bar chart showing the number of nodes (components) for the same groups as in panel C. (F) Scatter plot of NetMoss values comparing AH_AS and AH_AT, with labeled bacterial genera. Points above/below the diagonal line indicate increased/reduced abundance in AH_AS relative to AH_AT. (G) Scatter plot of NetMoss values comparing AH_AS and ATH_AT, highlighting bacterial genera. Adenoid hypertrophy (AH), adenotonsillar hypertrophy (ATH), nasopharyngeal swabs (NS), adenoid swabs (AS), tonsil swabs (TS), adenoid tissues (AT).

**Figure S6** **Comparative analysis of gene functional profiles across sample types and disease states.** (A) Heatmap illustrating the relative abundance of KEGG-annotated gene functions (rows) across sample types (Swab vs. Tissue) and disease states (Adenoid Hypertrophy, AH; Control; Tonsillar Hypertrophy, TH). Color gradient (blue: low, red: high) reflects normalized abundance values. Hierarchical clustering (Bray-Curtis distance) reveals distinct functional clusters associated with AH and TH. Pathways that significantly correlated with *Staphylococcus*, *Haemophilus* and *Streptococcus* (Spearman |R| > 0.3, adjusted *p* value < 0.05) are marked as red. (B-I) Boxplots quantifying the relative abundance (%) of eight selected gene functions (e.g., ko00650: Butanoate metabolism; ko00380: Tryptophan metabolism; ko00280: Valine, leucine and isoleucine degradation; ko00071: Fatty acid degradation) stratified by sample type and disease state. Significant differences (Mann-Whitney U test with Benjamini-Hochberg correction) are denoted with asterisks (*: *p* value < 0.05, **: *p* value < 0.01, ***: *p* value < 0.001, ****: *p* value < 0.0001). Sample types are color-coded (Swab: blue; Tissue: red), and disease states are differentiated by fill patterns (AH: diagonal stripes; Control: solid; TH: dots).

**Figure S7 Microscopic characterization and microbiological isolation of Staphylococcus aureus in infected adenoid tissues.** (A) Fluorescence microscopy of adenoid tissue sections stained with the universal bacterial probe EUB338 (green) and *S. aureus*-specific probes (red), counterstained with hematoxylin and eosin (HE). Representative images from two independent samples (S1P1, S1P2, S2P1 and S2P2) are shown at 200x and 400x magnifications. Highlighted regions (white boxes) indicate *S. aureus* microcolonies (arrows) and tissue architecture. (B) Schematic workflow for bacterial isolation and purification from adenoid tissues. Sequential saline washes (×5) were performed to collect loosely adherent bacteria (washing liquid), followed by mechanical homogenization of the adenoid core tissue. Isolated bacteria were cultured for 24 hours on agar plates and purified using standard microbiological methods. The reference strain S. aureus ATCC25923 was included for comparison. (C) Bacterial cultures from washing liquid (CUL) and adenoid core tissue (S1P1, S2P1) after 24-hour incubation. Colonies of S. aureus (yellow-white, β-hemolytic) are shown at 100x (colony morphology) and 400x (cellular purity) magnifications, demonstrating successful isolation from both compartments. (D) Representative culture plates from patients 30, 21, and 22 are displayed. For each patient, the upper row corresponds to adenoid washing liquid samples, while the lower row represents adenoid core tissue samples. Bacterial growth was assessed using selective agar medium specific for S. aureus (red agar), with distinct colonies indicative of *S. aureus* presence.

**Figure S8 Hierarchical clustering analysis of adenoid hypertrophy (AH) severity profiles across patient cohorts.** Hierarchical clustering heatmap of *Staphylococcus aureus* (A) and *Streptococcus pneumoniae* (B) in AH patient, with color-coded AH grade. Hierarchical clustering (left dendrogram, Bray-Curtis dissimilarity) groups patients based on AH grade similarity, with tree scale bar indicating phylogenetic distance. The same patients with two color squares means both adenoid tissue and swabs detected species*.*
